# Three-Dimensional Evaluation of the Effects of Different Treatment Methods on Pharyngeal Airways in Patients with Skeletal Class III Malocclusion

**DOI:** 10.3390/medicina61010142

**Published:** 2025-01-16

**Authors:** Mevlude Yuce Polat, İsmail Ceylan

**Affiliations:** 1 Department of Orthodontics, Faculty of Dentistry, Harran University, 63300 Sanliurfa, Turkey; 2 Department of Orthodontics, Faculty of Dentistry, Ataturk University, 25030 Erzurum, Turkey; iceylan@atauni.edu.tr

**Keywords:** skeletal Class III malocclusion, RME, FM, respiratory tract, CBCT, Dolphin software

## Abstract

*Background and Objectives*: The aim of this prospective study was to assess the effects of rapid maxillary expansion (RME) and/or face mask (FM) treatments on the pharyngeal airway in patients with skeletal Class III malocclusion caused by maxillary deficiency. This study utilized cone beam computed tomography (CIBT) for a three-dimensional (3D) analysis of airway changes, comparing the results with those of a control group consisting of untreated skeletal Class III patients. *Materials and Methods*: The study included 60 participants (34 boys, 26 girls) aged 9 to 14 years, all diagnosed with skeletal Class III malocclusion due to maxillary underdevelopment. The participants were divided into four treatment groups, each consisting of 15 individuals: Group 1—RME; Group 2—RME/FM; Group 3—FM; Group 4—Control group. The pharyngeal airway measurements were evaluated using CBCT and analyzed with Dolphin 3D software (version 11.9). Volumetric parameters and minimal axial area (MAA) were measured in the nasopharyngeal, retropalatal, retroglossal, and total airway regions. The collected data were statistically analyzed using SPSS 20.0 software. *Results*: The results indicated significant changes in pharyngeal airway volumes across all treatment groups compared to the control group. A statistically significant increase in nasopharyngeal, retropalatal, and total airway volume were observed in all treatment groups. Only the RME group demonstrated a significant increase in retroglossal volume. Significant increases in MAA were found in the RME/FM and FM groups in the nasopharyngeal and retropalatal regions. However, minimal changes were observed in the retroglossal region across all treatment groups. The control group showed no significant changes in any of the measured parameters, underscoring the effects of the treatments. *Conclusions*: The findings of this study suggest that both RME and/or FM treatments result in significant positive changes in the pharyngeal airways, particularly in the nasopharyngeal and retropalatal regions. The retroglossal region showed more limited response to the treatments. The combined RME/FM therapy was found to be particularly effective in improving airway dimensions in the anterior and mid-pharyngeal regions. These results highlight that early orthodontic interventions, such as RME and FM, can improve both airway patency and overall respiratory function, in addition to addressing skeletal Class III malocclusion.

## 1. Introduction

Although skeletal Class III malocclusions are less common than other malocclusions, their differential diagnosis and treatment are more difficult. These anomalies can develop as a combination of a normal upper jaw and mandibular advancement, a normal lower jaw and mandibular retardation, or mandibular retardation and mandibular advancement [1].

Class III anomalies due to maxillary insufficiency can usually be treated with orthopedic appliances such as functional treatment devices like Frankel or FM, but at later ages they can be treated with fixed orthodontic techniques and surgical treatment techniques [2,3]. Orthodontic treatment aims to enhance esthetic appearance and achieve a more balanced occlusion through changes in the dental, maxillofacial, and facial structures, while also addressing existing issues in the functions of the stomatognathic system, such as chewing, speaking, and breathing. With orthopedic and orthognathic surgical treatment approaches, significant changes can be created especially in the bony structures surrounding the upper airway, pharyngeal soft tissues, tongue, and hyoid bone. As a result of these changes, a significant improvement in respiratory function can be achieved [4,5]. The literature suggests that a posteriorly positioned upper jaw is associated with a tendency to narrow the airway dimensions, while forward development and more anterior positioning of the upper jaw have a positive impact on the size of the upper airway [5,6]. In this respect, the dentofacial effects of RME, especially its effects on the upper respiratory tract, have been the subject of numerous studies [5,7,8].

The face mask, which aims to cause a skeletal protraction of this structure in a block by stimulating maxillary development, is widely used in the early orthopedic treatment of Class III malocclusions due to maxillary retrusion [9]. RME with the use of a FM has been reported to correct posterior crossbite by providing transversal expansion of the maxilla and to facilitate anterior movement of the maxilla by stimulating the circummaxillary sutures.

Although numerous studies have examined the dental and/or skeletal effects of orthopedic and surgical treatments aimed at correcting the position of the upper and lower jaws and their relationship, studies investigating changes in pharyngeal airway dimensions are relatively limited [10,11]. However, there is a notable lack of studies that examine this topic using three-dimensional imaging and evaluation methods [7,12,13].

The aim of this prospective study was to investigate changes in airway dimensions in individuals with skeletal Class III malocclusion due to maxillary developmental insufficiency, treated with RME and/or FM, using 3D imaging and assessment techniques, and to compare the results with those obtained from an untreated control group.

## 2. Materials and Methods

This prospective study was reviewed and approved by the Ethics Committee of the Faculty of Dentistry, Ataturk University, Erzurum, Turkey. The study was deemed to comply with ethical standards, as confirmed by the decision report of the relevant board, with reference number 07-2015-37. Furthermore, all participants and their families were thoroughly informed about the orthodontic treatment to be applied, alternative treatment options were presented, and informed consent forms were obtained from both the patients and their parents

The study group comprised 60 individuals, including 34 males and 26 females, all with growing skeletal Class III malocclusion due to maxillary insufficiency, who sought treatment at the Department of Orthodontics, Faculty of Dentistry, Ataturk University, Erzurum, Turkey. The participants were divided into four groups, each consisting of 15 subjects: Group 1 (RME) included those who underwent rapid maxillary expansion, Group 2 (RME/FM) included those who received both rapid maxillary expansion and face mask therapy, Group 3 (FM) consisted of individuals treated with FM alone, and Group 4 (Control) comprised those who did not receive any treatment.

The inclusion criteria for participants in the study were as follows: normal growth and developmental patterns, presence of skeletal Class III malocclusion due to maxillary underdevelopment and/or maxillary constriction, SNA angle less than 78°, ANB angle less than 0°, Wits value less than −1 mm, chronological age between 9 and 14 years, presence of a head-to-head or crossbite relationship between the incisors, normal upright growth pattern, being in a phase of active growth and development. All participants and their families provided informed consent prior to participation. The exclusion criteria for participants in the study were as follows: a history of any systemic diseases or trauma that could adversely affect growth and development, congenital or genetic craniofacial deformities such as cleft lip and palate, known respiratory or sleep disorders, or any airway pathologies, history of adenoidectomy or tonsillectomy, previous orthodontic treatment, any congenital tooth anomalies or tooth extractions that could impact the size of the maxilla, abnormal tongue function or swallowing patterns. Participants with psychological or behavioral conditions that could interfere with treatment compliance were excluded. Participants with horizontal or atypical growth patterns were excluded from the study. Participants and their families were informed about the necessity and safety of CBCT imaging, and its use was strictly limited to essential assessments for the study.

**Figure 1:**
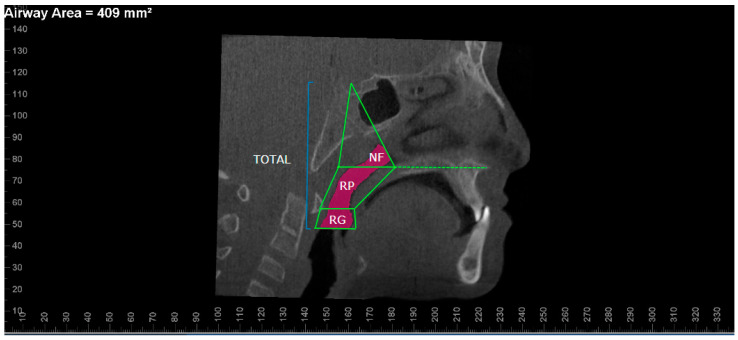
Boundaries and volume measurements used in this study. (NF—nasopharyngeal, RP—retropharyngeal, RG—retroglossal).

**Figure 2:**
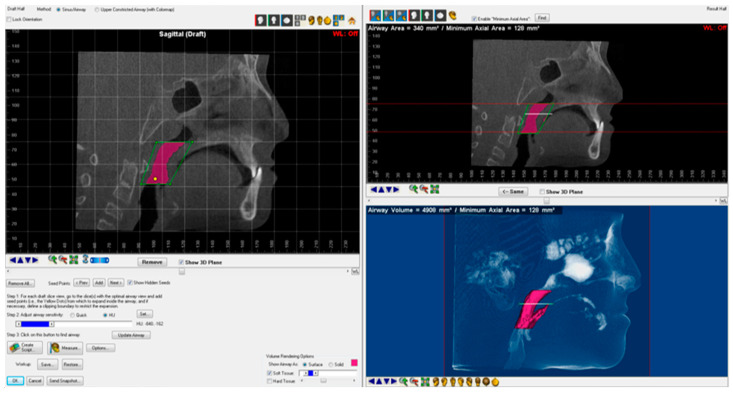
Boundaries and measurements of MAA (minimal axial area) used in this study.

All participants were evaluated at baseline (T1) and at subsequent time points: 3 months after RME completion (T2) for RME group, 6 months after maxillary expansion and protraction (T2) for RME/FM and FM groups, and 6 months post-treatment (T2) for control group. CBCT images were acquired using 0.5 mm axial slices in DICOM format with a NewTom 3G FPI (QR-DVT-9000, QR srl, Verona, Italy). Additionally, intraoral and extraoral photographs, orthodontic models, digital panoramic radiographs, hand-wrist films, and posteroanterior and lateral cephalometric radiographs were obtained at the same time points. The CBCT images in DICOM format were imported into Dolphin 3D (Version 11.9, Dolphin Imaging & Management Solutions, Chatsworth, CA, USA) for 3D reconstruction. The orientation process was performed as follows: [13,14]. In the frontal view, the skeletal midline (N-ANS) was positioned perpendicular to the ground, with the right and left orbital points parallel to the ground. In the axial view, the midsagittal plane (from the incisive foramen to the opisthion) was made perpendicular to the ground. In the midsagittal view, the coronal plane was oriented to pass through the furcation level of the upper right first molar and perpendicular to the Frankfort horizontal plane (FH). For the accuracy of airway measurements, after selecting the PNS and ANS points on the midsagittal image, the palatal plane was reoriented so that it was parallel to the ground, and the other planes were adjusted parallel to the palatal plane. The NF, RP, and RG airway segments and measurements used in the study are shown in Figure 1. MAA boundaries and measurement are shown in Figure 2.

The Hyrax screw (Leone, Italy), in the RME and RME/FM groups, allows for up to 11 mm of expansion, with each quarter turn providing 0.25 mm of expansion. During the first week, patients were instructed by their parents to turn the screw by one-quarter turn every morning and evening. One week later, occlusal radiographs confirmed separation of the midpalatal suture, and the expansion was continued with a daily quarter-turn. Active expansion was discontinued once the posterior crossbite was corrected, and the screw was stabilized with a ligature wire. After a 3-month retention period, the second CBCT recordings were obtained.

In the RME/FM group, the Hyrax screw was activated with two quarter turns per day during the first week, followed by the application of a Petit-type FM. Expansion continued until sufficient transverse expansion was achieved, with 400 g of protraction force applied on each side using elastics. The FM was adapted to the patient’s face, and patients were instructed to wear it for 16–18 h daily. Treatment was continued for a total of 6 months, until a Class II molar and canine relationship, along with a 2–5 mm overjet and convex profile, were achieved. In the RME/FM and FM groups, a Petit-type FM (Protraction Face Mask, G&H Wire Company, Franklin, IN, USA) was used. In the control group (Group 4), no treatment was applied, and re-recordings were taken after a 6-month observation period.

### Statistical Analysis

Statistical analysis was performed using IBM SPSS Statistics version 20.0 (Statistical Package for Social Sciences). Descriptive statistics, including minimum, maximum, mean, and standard deviation, were calculated for all parameters. The Shapiro–Wilk test was applied to assess the normality of the data distribution.

For comparisons between two independent groups, the independent samples *t*-test was used for normally distributed parameters, while the Mann–Whitney U test was employed for non-normally distributed parameters. In intra-group comparisons, the paired samples *t*-test was used for parameters with a normal distribution, and the Wilcoxon signed-rank test was applied for non-normally distributed parameters.

For intergroup comparisons of continuous variables across more than two groups, the one-way ANOVA test was used for normally distributed variables, and the Kruskal–Wallis test was applied for non-normally distributed variables. When significant intergroup differences were found following the ANOVA test, the Tukey’s test was used for post hoc comparisons when variances were homogeneous, and the Tamhane’s T2 test was used when variances were heterogeneous.

For comparisons between categorical variables, the Chi-square test and Fisher’s Exact test were employed. A statistical significance level of *p* < 0.05 was considered significant.

To assess the repeatability of the three-dimensional airway measurements, all volumetric, areal, and linear measurements were repeated approximately 1 month after the initial measurements on the KIBT records of 20 randomly selected patients. The method error was evaluated by calculating the intraclass correlation coefficient (ICC), with the results assessed using a 95% confidence interval.

While no significant difference was observed between the groups in terms of chronological age at the beginning of treatment, a significant difference in treatment duration was found between the RME group and the other groups (*p* < 0.001). Descriptive statistics, ANOVA, and Kruskal–Wallis test results, along with *p*-values for certain cephalometric data at the beginning of treatment or observation (T1), are presented in Table 1.

## 3. Results

The Chi-square test results and *p*-values for the number and gender distribution of individuals across all groups are presented in Table 2, along with the differences between the groups. No significant differences were observed between the groups in terms of the number of individuals or gender distribution. All within-group and between-group statistical comparisons were based on the differences observed between T1 (pre-treatment) and T2 (post-treatment or post-observation). Paired sample *t*-tests (for normally distributed parameters) and Wilcoxon tests (for non-normally distributed parameters) were applied to evaluate the statistical significance of the differences before and after treatment for all measurements.

### 3.1. Intra-Group Comparisons

All statistical tests were conducted to assess the significance of the differences before and after treatment, with the results summarized in Table 3, Table 4, Table 5 and Table 6.

In the RME group, significant increases were observed in nasopharyngeal, retroglossal, and total pharyngeal volumes (*p* < 0.001), as well as in retropalatal volume (*p* < 0.01). However, no significant change was found in the retroglossal volume and MAA (Table 3).

In the RME/FM group, significant increases were observed in the retropalatal volume (*p* < 0.01), nasopharyngeal volume, and total pharyngeal volume (both at *p* < 0.001). No significant change was noted in the retroglossal volume. Additionally, a significant increase was found in the minimal axial area (MAA) at the *p* < 0.05 level (Table 4).

In the FM group, significant increases were observed in nasopharyngeal, retropalatal, and total pharyngeal volumes (*p* < 0.001), while no significant change was detected in the retroglossal volume measurement. Additionally, a significant increase was found in MAA at the *p* < 0.01 level (Table 5).

No statistically significant changes were observed in any of the measurements in the control group (Table 6).

### 3.2. Inter-Group Comparisons

Descriptive statistical values for the comparison of the differences between T1 and T2 between the groups and the results of the ANOVA (for normally distributed parameters) and Kruskal–Wallis test (for non-normally distributed parameters) and post hoc Tukey (for normally distributed parameters) and Tamhanes T2 (for non-normally distributed parameters) tests, applied to determine the significance levels of the differences, are given in Table 7.

As a result of the comparison of intergroup differences, significant intergroup differences were found in nasopharyngeal, retropalatal, and total pharyngeal volume measurements at *p* < 0.001 level, while no significant difference was found in retroglossal volume measurement. In conclusion, the analysis revealed significant intergroup differences in nasopharyngeal, retropalatal, and total pharyngeal volumes, with all treatment groups showing improvements compared to the control group. However, retroglossal volume did not exhibit significant changes across the groups, and differences in nasopharyngeal volume were specifically noted between the RME and FM groups (Table 7).

## 4. Discussion

The relationship between pharyngeal and craniofacial structures has been extensively investigated in the literature [5,7,11,15,16,17,18]. Research has indicated that posterior positioning of the upper and lower jaws can contribute to a reduction in the anteroposterior dimensions of the airway [19]. Numerous studies focused on the clinical characteristics of Class III malocclusions have demonstrated that these malocclusions are predominantly associated with maxillary underdevelopment [1]. Furthermore, it has been reported that skeletal Class III cases, characterized by maxillary underdevelopment, have a higher incidence of upper airway obstruction. Early treatment aimed at promoting the development of the maxilla in both the sagittal and transverse directions has been shown to have a positive effect on the dimensions of the upper airway [6,8,9]. Methods such as cephalometry, MRI, CT, and KIBT are frequently employed in the radiographic evaluation of the respiratory tract [20]. Numerous studies have demonstrated that KIBT offers significant advantages over traditional two-dimensional radiographs, particularly in overcoming issues such as distortion, superposition, and magnification. Due to its low cost, minimal radiation exposure, and ease of application, KIBT has been recognized as a highly reliable and effective method for assessing the pharyngeal airway [20,21].

In our study, the segmentation of the pharyngeal airway was conducted separately for the nasopharyngeal, retropalatal, retroglossal, and total volumes, following a methodology similar to that of Pliska et al. [14]. The literature shows considerable variation in both the number of points and planes used for airway segmentation, as well as the number of segmentation regions employed [7,13,14]. In our study, the selection of points for evaluating the dimensions of the pharyngeal airways was based on criteria such as ease of reproducibility, sensitivity to changes in pharyngeal tissues, and consistency with recent studies [14,22].

Although RME is not typically considered one of the primary treatment modalities for skeletal Class III malocclusion, it plays a crucial supportive role. Since skeletal Class III malocclusions are often characterized by maxillary constriction and retrusion, RME can be an effective treatment option to address maxillary stenosis, thereby enhancing the outcomes of maxillary protraction therapy [23]. Given that the maxilla anatomically forms the floor of the nasal cavity and the anterior boundary of the nasopharyngeal airway, studies have indicated that widening the maxilla leads to an increase in both the floor of the nasal cavity and the width of the nasopharynx. Additionally, forward movement of the maxilla has been shown to enhance the sagittal dimensions of the nasopharyngeal airway [24].

In our study, nasopharyngeal, retropalatal and total pharyngeal volume increased statistically significantly in all treatment groups, while no significant change was observed in the control group. A statistically significant increase in retroglossal airway volume was found only in the RME group. These findings indicate that the treatment methods were effective in increasing the dimensions of the pharyngeal airway, but the effect was more pronounced in the upper regions (nasopharyngeal and retropalatal) and less in the lower regions (retroglossal). The lack of a significant increase in retroglossal volume, except in the RME group, may also be attributed to a decrease in airway dimensions in the relevant region due to the downward rotation of the lower jaw.

Comparisons between the groups showed that the RME group achieved approximately twice the increase in nasopharyngeal volume compared to the face mask group (970 mm³ vs. 490 mm³). This finding suggests that RME is a more effective method of expanding the nasopharyngeal airway than a face mask. The significant increase in nasopharyngeal, retropalatal, and total pharyngeal volume in the treatment groups compared to the control group indicates that the treatment methods (RME and/or FM) are highly effective in increasing airway dimensions, independent of the effects of growth and development. It can be said that this change is a result of the increase in the oropharyngeal area and the more anterior positioning of the tongue, especially with the expansion and forward movement of the upper jaw [9]. The lack of significant difference in retroglossal volume between the groups may indicate that the treatment effects decrease towards the lower parts of the pharyngeal airway.

The findings of our study align with the existing literature, which reports an increase in pharyngeal airway volume following RME and/or FM treatment [4,6,25]. Notably, several studies have demonstrated that RME is effective in increasing airway volume [7,13]. However, it should be noted that the majority of these studies, particularly those involving the use of face masks, are cephalometric studies in which the evaluated structures are examined in two dimensions [6,25]. Furthermore, it was observed that in many of these studies, the study group was compared to a cross-sectional control group, rather than to a longitudinal examination of individuals with malocclusion similar to that of the treatment groups in our study [4,26].

In the study by El and Palomo [7], significant increases in nasal passage and oropharyngeal volumes were observed following RME; however, it was noted that the increase in oropharyngeal volume was attributed to growth. Similarly, other studies on RME have reported that soft tissues follow bone expansion, with significant increases in nasopharyngeal airway volume [13]. Additionally, these studies also reported an increase in nasopharyngeal volume after RME, but a non-significant decrease in oropharyngeal volume due to changes in the palatal plane [13]. These findings are consistent with the increases in nasopharyngeal and retropalatal volumes observed in our study. However, the changes in oropharyngeal volume were more limited. The restricted volume changes in the retroglossal region in our study suggest that the effect of the treatment methods diminishes as one moves toward these lower regions. In agreement with this, Chang et al. [22] reported that the effect of RME on the upper airway is local and likely the result of soft tissue adaptation. Furthermore, this effect diminishes as one moves further from the maxillary suture, particularly toward the lower sections of the pharyngeal airway.

Contrary to our findings, some studies have reported no significant changes in the upper, lower, or total pharyngeal airway volumes following RME and/or FM treatment [27]. This discrepancy may be attributed to methodological differences between the studies, as well as factors such as age group and treatment duration. Additionally, it is important to note that regional volume measurements, particularly in the lower regions of the pharyngeal airways, may be less reliable than total volume measurements. This is due to differences in segmentation methods and the involvement of soft tissue functional movements, which can lead to varying results across studies.

In the assessment of pharyngeal airways, measuring the MAA is crucial, as narrowing of this region may increase the risk of obstructive sleep apnea syndrome [28]. We believe that the observed improvements in nasopharyngeal and retropalatal airway volumes in this study may contribute to reducing the risk of clinical conditions, such as obstructive sleep apnea (OSA), which are commonly associated with airway constriction. It has been reported that the size and shape of the MAA can vary depending on positional changes in the mandible, tongue, or soft palate, and that different treatment modalities may produce distinct changes in this area [29]. In our study, the increase in the MAA was not statistically significant in the RME group, whereas significant increases were observed in the RME/FM and FM groups. No change was noted in the control group. It is hypothesized that the FM application positively influences the retropalatal region through maxillary advancement. However, no significant differences were found between the groups in terms of MAA. Chen et al. [4] reported significant increases in the volume of the nasopharyngeal and oropharyngeal regions following RME and FM treatment, which were accompanied by an increase in the MAA in these regions. However, no significant changes were observed in the hypopharyngeal region. These findings are consistent with the results of our study. Zhao et al. [30] did not report any significant changes in the MAA following RME.

In this study, obtaining CBCT images from individuals in the control group at 6-month intervals may provoke discussion in terms of ethics and radiation exposure. However, this practice necessitated the existence of a control group in order to evaluate the effects of the treatment groups independent of growth and development. The main purpose of obtaining CBCT images was explained to the patients and their families in the control group in detail. The main purpose of obtaining CBCT images was not only to monitor post-treatment changes, but also to help plan the timing of skeletal Class III malocclusion due to maxillary deficiency during treatment.

## 5. Conclusions

In conclusion, RME and FM therapy are effective in increasing pharyngeal airway dimensions, with RME being more effective than FM therapy in enhancing nasopharyngeal airway volume. The findings of our study suggest that orthopedic treatment approaches applied to individuals with skeletal Class III malocclusion due to maxillary underdevelopment have a positive impact on the dimensions of the upper airways in the short term. However, it is essential to investigate whether these treatment effects are sustained in the long term. Furthermore, additional research is needed to explore how changes in airway dimensions following treatment may influence long-term respiratory function. Given that airway dimensions are a dynamic function shaped by various factors, future studies should not only focus on volumetric measurements but also include functional assessments. Methods such as polysomnography, acoustic rhinometry, and rhinomanometry should be utilized to examine the effects of these treatment modalities on respiratory function in greater detail.

## Figures and Tables

**Table 1:** Selected demographic and cephalometric data.

	Patient Groups																			
	Group 1: RME				Group 2: RME/FM				Group 3: FM				Group 4: Control							
	Mean (M)	Std. Dev. (SD)	Min	Max	Mean (M)	Std. Dev. (SD)	Min	Max	Mean (M)	Std. Dev. (SD)	Min	Max	Mean (M)	Std. Dev. (SD)	Min	Max	F	Chi-Square	p	Post Hoc
Age (years)	12.3	1.3	10.0	13.7	12.1	1.3	9.8	13.8	11.8	1.1	9.1	13.2	11.9	1.1	9.1	13.3	-	2.132	0.546	-
Duration (months)	4.11	0.19	3.80	4.40	6.33	0.36	5.30	6.90	6.08	0.48	5.00	6.70	6.01	0.15	5.80	6.40	149.238	-	0.000	1–2, 1–3, 1–4
SNA (°)	76.2	1.4	72.7	77.9	76.0	1.3	73.7	77.5	76.2	1.2	73.5	77.5	75.7	1.6	72.7	74.7	-	0.557	0.906	-
SNB (°)	77.9	1.8	73.5	81.2	78.6	1.5	75.6	81.1	78.3	1.6	74.9	80.7	77.3	1.8	74.6	81.1	1.658	-	0.187	-
ANB (°)	−1.6	1.0	−3.6	−0.1	−2.3	1.1	−4.0	−0.3	−2.2	1.1	−3.5	−0.1	−1.5	1.2	−3.9	0.5	2.119	-	0.108	-

F—ANOVA test, Chi-square, Kruskal–Wallis test. -: Not significant.

**Table 2:** Gender distribution.

			Patient Groups				Total	Test	
			RME	RME/FM	FM	Control		Chi-Square	*p*
Gender	Male	N	9	7	10	8	34	1.357	0.716
		%	26.5%	20.6%	29.4%	23.5%	100.0%		
	Female	N	6	8	5	7	26		
		%	23.1%	30.8%	19.2%	26.9%	100.0%		
Total		N	15	15	15	15	60		
		%	25.0%	25.0%	25.0%	25.0%	100.0%		

**Table 3:** Comparison of pre- and post-treatment airway volume values in the RME group.

Measurement (mm^3^)		Mean	Std. Dev.	Min.	Max.	t	Z	*p*	Significance
RME (*n* = 15)	NF t1	3640.27	1480.17	833.00	6059.00	−6.625	-	0.000	***
	NF t2	4610.93	1638.15	1775.00	6677.00				
	RP t1	5075.67	1326.04	3252.00	8327.00	-	−3.408	0.001	**
	RP t2	5691.60	1362.94	3531.00	8656.00				
	RG t1	3533.40	1570.50	1784.00	7990.00	−4.909	-	0.000	***
	RG t2	3704.20	1611.87	1822.00	8322.00				
	Total t1	12,249.33	3035.76	8049.00	17,737.00	−7.496	-	0.000	***
	Total t2	14,006.73	2872.72	9846.00	18,454.00				
	MAA t1	130.73	38.18	74.00	200.00	−2.018	-	0.063	-
	MAA t2	145.93	47.99	85.00	238.00				

t—paired samples *t*-test, Z—Wilcoxon test, *n*—number of individuals. Significance levels: **: *p* < 0.01, ***: *p* < 0.001.

**Table 4:** Comparison of pre- and post-treatment airway volume values in the RME + Facemask group.

Measurements (mm^3^)		Mean	Std. Dev.	Min.	Max.	t	Z	*p*	Significance
RME/FM (*n* = 15)	NF t1	2725.07	950.00	1463.00	4492.00	−6.994	-	0.000	***
	NF t2	3611.60	892.65	2471.00	5126.00				
	RP t1	4778.87	1396.46	3115.00	8365.00	−3.924	-	0.002	**
	RP t2	5654.13	1715.36	3263.00	9253.00				
	RG t1	3281.67	1036.81	1556.00	5096.00	−1.960	-	0.070	-
	RG t2	3440.40	1157.18	1439.00	5787.00				
	Total t1	10,785.60	2254.40	7104.00	15,187.00	−5.959	-	0.000	***
	Total t2	12,706.13	2866.43	7578.00	17,819.00				
	MAA t1	119.40	47.63	44.00	225.00	−2.627	-	0.020	*
	MAA t2	138.73	57.10	55.00	215.00				

t—paired samples *t*-test, Z—Wilcoxon test, *n*—number of individuals. Significance levels: *: *p* < 0.05, **: *p* < 0.01, ***: *p* < 0.001. -: Not significant.

**Table 5:** Comparison of pre- and post-treatment airway volume values in the Facemask group.

Measurements (mm^3^)		Mean	Std. Dev.	Min.	Max.	t	Z	*p*	Significance
Yüz maskesi (*n* = 15)	NF t1	3234.80	1389.42	1625.00	7256.00	−6.930	-	0.000	***
	NF t2	3725.60	1401.49	1982.00	7557.00				
	RP t1	5344.60	1999.84	2960.00	8751.00	−11.447	-	0.000	***
	RP t2	6177.47	2000.41	3506.00	9873.00				
	RG t1	3022.20	1294.50	1463.00	5611.00	−0.958	-	0.354	-
	RG t2	3149.67	1247.43	1308.00	5899.00				
	Total t1	11,601.60	3487.83	7219.00	20,170.00	−8.924	-	0.000	***
	Total t2	13,052.73	3418.30	8786.00	21,462.00				
	MAA t1	133.13	69.38	66.00	317.00	−3.498	-	0.004	**
	MAA t2	149.47	62.15	79.00	321.00				

t—paired samples *t*-test, Z—Wilcoxon test, *n*—number of individual. Significance levels: **: *p* < 0.01, ***: *p* < 0.001. -: Not significant.

**Table 6:** Comparison of pre- and post-observation airway volume values in the Control group.

Measurements (mm^3^)		Mean	Std. Dev.	Min.	Max.	t	Z	*p*	Significance
Control (*n* = 15)	NF t1	2707.53	759.24	1800.00	4982.00	−0.697	-	0.497	-
	NF t2	2765.13	762.03	1707.00	4826.00				
	RP t1	4040.07	1072.88	2213.00	6180.00	0.720	-	0.483	-
	RP t2	3960.27	1029.28	2510.00	6577.00				
	RG t1	2166.60	762.39	1380.00	4349.00	−0.646	-	0.529	-
	RG t2	2244.27	768.44	1235.00	3999.00				
	Total t1	8914.20	1899.15	6162.00	11,761.00	−0.262	-	0.797	-
	Total t2	8969.67	1636.38	6172.00	11,884.00				
	MAA t1	114.33	35.76	55.00	172.00	−0.399	-	0.696	-
	MAA t2	116.67	36.50	67.00	207.00				

t—paired samples *t*-test, Z—Wilcoxon test, *n*—number of individuals. -: Not significant.

**Table 7:** Intergroup comparisons of differences in pre- and post-treatment volume values.

	Patient Groups																			
	RME				RME/FM				FM				Control							
	Mean	Std. Dev.	Min.	Max.	Mean	Std. Dev.	Min.	Max.	Mean	Std. Dev.	Min.	Max.	Mean	Std. Dev.	Min.	Max.	F	Chi-Square	*p*	Post Hoc
t2–1 NF (mm^3^)	970.67	567.45	120.00	2260.00	886.53	490.90	203.00	1865.00	490.80	274.30	89.00	1066.00	57.60	320.04	−349.00	690.00	14.193	-	0.000	1–3, 1–4, 2–4, 3–4
t2–t1 RP (mm^3^)	615.93	492.87	80.00	1536.00	875.27	863.99	−170.00	2523.00	832.87	281.80	408.00	1278.00	−79.80	429.08	−830.00	719.00	-	22.631	0.000	1–4, 2–4, 3–4
t2–t1 RG (mm^3^)	170.80	134.76	−43.00	399.00	158.73	313.72	−278.00	691.00	127.47	515.27	−985.00	995.00	77.67	465.73	−774.00	994.00	0.173	-	0.914	-
t2–t1 Total (mm^3^)	1757.40	908.05	621.00	3437.00	1920.53	1248.13	252.00	4098.00	1451.13	629.78	144.00	2438.00	55.47	821.14	−1020.0	1649.0	12.544	-	0.000	1–4, 2–4, 3–4
t2–t1 MAA (mm^2^)	15.20	29.17	−31.00	88.00	19.33	28.50	−16.00	79.00	16.33	18.09	−28.00	56.00	2.33	22.64	−29.00	44.00	1.354	-	0.266	-

F—ANOVA test, Chi-square, Kruskal–Wallis test. -: Not significant.

## Data Availability

The data presented in this study are available on request from the corresponding author.
